# Early warning signals in psychopathology: what do they tell?

**DOI:** 10.1186/s12916-020-01742-3

**Published:** 2020-10-14

**Authors:** Marieke J. Schreuder, Catharina A. Hartman, Sandip V. George, Claudia Menne-Lothmann, Jeroen Decoster, Ruud van Winkel, Philippe Delespaul, Marc De Hert, Catherine Derom, Evert Thiery, Bart P. F. Rutten, Nele Jacobs, Jim van Os, Johanna T. W. Wigman, Marieke Wichers

**Affiliations:** 1 Department of Psychiatry, Interdisciplinary Center Psychopathology and Emotion regulation (ICPE), University of Groningen, University Medical Center Groningen, Internal Postal Code: CC72, Triade Building Entrance 24, Hanzeplein 1, 9713 GZ Groningen, The Netherlands; 2 Department of Psychiatry and Neuropsychology, School of Mental Health and Neuroscience (MHeNS), Maastricht University, Universiteitssingel 40, 6299 ER Maastricht, The Netherlands; 3 University Psychiatric Centre, KU Leuven, Herestraat 49, 3000 Leuven, Belgium; 4 Department of Neurosciences, Center for Public Health Psychiatry, KU Leuven, Herestraat 49, 3000 Leuven, Belgium; 5 Department of Neurosciences, Center for Clinical Psychiatry, KU Leuven, Kapucijnenvoer 7, 3000 Leuven, Belgium; 6 Mondriaan Mental Health Care, John F. Kennedylaan 301, 6419 XZ Heerlen, The Netherlands; 7 Antwerp Health Law and Ethics Chair – AHLEC, University of Antwerp, Antwerp, Belgium; 8 Centre of Human Genetics, University Hospital Leuven, KU Leuven, Herestraat 49, 3000 Leuven, Belgium; 9 Department of Obstetrics and Gynecology, Ghent University Hospital, Ghent University, C. Heymanslaan 10, 9000 Ghent, Belgium; 10 Department of Neurology, Ghent University Hospital, Ghent University, C. Heymanslaan 10, 9000 Ghent, Belgium; 11 Faculty of Psychology and Educational Sciences, Open University of the Netherlands, Valkenburgerweg 177, 6419 AT Heerlen, The Netherlands; 12 Department of Psychosis Studies, Institute of Psychiatry, King’s Health Partners, King’s College London, De Crespigny Park, London, SE5 8AF UK; 13 Department Psychiatry, Brain Center Rudolf Magnus,, Utrecht University Medical Centre, Universiteitsweg 100, 3584 CG Utrecht, The Netherlands

**Keywords:** Early warning signals, Momentary affective states, Complex systems, Symptom development, Psychopathology

## Abstract

**Background:**

Despite the increasing understanding of factors that might underlie psychiatric disorders, prospectively detecting shifts from a healthy towards a symptomatic state has remained unattainable. A complex systems perspective on psychopathology implies that such symptom shifts may be foreseen by generic indicators of instability, or early warning signals (EWS). EWS include, for instance, increasing variability, covariance, and autocorrelation in momentary affective states—of which the latter was studied. The present study investigated if EWS predict (i) future worsening of symptoms as well as (ii) the type of symptoms that will develop, meaning that the association between EWS and future symptom shifts would be most pronounced for congruent affective states and psychopathological domains (e.g., feeling down and depression).

**Methods:**

A registered general population cohort of adolescents (mean age 18 years, 36% male) provided ten daily ratings of their affective states for 6 consecutive days. The resulting time series were used to compute EWS in feeling down, listless, anxious, not relaxed, insecure, suspicious, and unwell. At baseline and 1-year follow-up, symptom severity was assessed by the Symptom Checklist-90 (SCL-90). We selected four subsamples of participants who reported an increase in one of the following SCL-90 domains: depression (*N* = 180), anxiety (*N* = 192), interpersonal sensitivity (*N* = 184), or somatic complaints (*N* = 166).

**Results:**

Multilevel models showed that EWS in feeling suspicious anticipated increases in interpersonal sensitivity, as hypothesized. EWS were absent for other domains. While the association between EWS and symptom *increases* was restricted to the interpersonal sensitivity domain, post hoc analyses showed that symptom severity at baseline was related to heightened autocorrelations in congruent affective states for interpersonal sensitivity, depression, and anxiety. This pattern replicated in a second, independent dataset.

**Conclusions:**

The presence of EWS prior to symptom shifts may depend on the dynamics of the psychopathological domain under consideration: for depression, EWS may manifest only several weeks prior to a shift, while for interpersonal sensitivity, EWS may already occur 1 year in advance. Intensive longitudinal designs where EWS and symptoms are assessed in real-time are required in order to determine at what timescale and for what type of domain EWS are most informative of future psychopathology.

## Background

Psychiatric disorders affect approximately 1 in 4 individuals at some point in their lives [1]. Given the burden associated with such disorders, preventing their onset and progression may substantially improve individuals’ well-being. Traditionally, the quest for prevention has been pursued through understanding the risk factors and mechanisms that may give rise to psychopathology [2]. At present, numerous risk factors have been proposed. Yet, the complexity of the mechanisms by which they contribute to psychopathology challenges accurate identification of who is when at risk for developing symptoms. This calls for a novel perspective on psychopathology.

A complex systems perspective may answer this call [3–5]. According to this perspective, sudden shifts—including increases in psychiatric symptoms—may be understood regardless of our limited insight in the mechanisms that cause them. The underlying premise is that psychopathology can be described by distinct equilibrium states featured by absent/mild or severe symptoms, respectively. Shifts between these equilibria, marking the onset or remittance of symptoms, may occur in an abrupt, stepwise fashion rather than gradually [6, 7]. The likelihood of such shifts depends on the system’s stability [8]. In a stable system, temporary departures from equilibrium caused by small environmental perturbations are met with regulatory processes that quickly restore the equilibrium. In other words, stressful events (perturbations) may cause brief increases in symptoms (departure from equilibrium), followed by remission of these symptoms (return to equilibrium). As instability increases, the system’s resilience against perturbations declines, and consequently, a shift towards another equilibrium (e.g., presence of symptoms) becomes more likely [9, 10]. Exposing a system’s instability might thus allow us to foresee sudden shifts from absent/mild to more severe symptoms.

Empirical support for complex systems principles in psychopathology was provided by studies that confirmed that markers of instability—specifically, critical fluctuations—indeed predict symptom shifts [11–13]. The present paper does not discuss critical fluctuations [8, 11, 14–16], but rather focuses on critical slowing down as a marker of instability. Critical slowing down refers to an increasingly slowed return to equilibrium [10, 17]. Because of their ability to warn for an upcoming shift to an alternate equilibrium state, indicators of critical slowing down have been referred to as early warning signals (EWS) [10]. EWS include, for instance, increasing variability, covariance, and autocorrelation, as well as multivariate extensions of such metrics [18]. Because autocorrelations are relatively robust EWS and have been established previously in observational, psychological data [4, 5, 19], the remainder of this article will focus on this metric^1^ [9, 10, 17, 19]. In the context of psychopathology, autocorrelations refer to the degree to which an individual’s current affective state (e.g., feeling down) is predictive of his/her future affective state [4, 20–22]. When autocorrelations are high, temporary rises in an affective state in response to a stressful event (e.g., feeling down after failing a test) persist over time. This resistance to change in affective states prevents the system from quickly returning to its equilibrium state (e.g., absence of symptoms, and hence, not feeling down) [20, 23–25]. A complex systems perspective on psychopathology thus proposes that elevated autocorrelations in momentary affective states might warn for an upcoming shift in symptoms. Such shifts can theoretically reflect either a decrease or an increase in symptoms [4], although most research so far has focused on increases in symptoms.

Earlier studies have provided indirect support for EWS in psychopathology by showing that increased autocorrelations in affective states might cross-sectionally relate to several maladaptive characteristics, including low self-esteem [24, 25], neuroticism [23], and the presence of psychiatric disorders [20, 21, 24, 26, 27]. These studies raised two compelling questions. Specifically, it remained unclear (i) whether autocorrelations in affective states also prospectively predict psychopathology—as would be expected from complex systems principles, and (ii) whether the predictive utility of autocorrelations extends beyond that of mean affect levels [28].

Initial support for the prospective association between EWS—operationalized by elevated autocorrelations in affective states—and symptom shifts was provided by van de Leemput and colleagues [4], who demonstrated that individuals with higher EWS in affective states, such as feeling down and anxious, later reported higher depressive symptom severity. EWS were inferred from momentary ratings of affective states acquired through the experience sampling method (ESM). These results were in line with what would be expected from complex systems principles, but did not prove the presence of EWS prior to worsening symptoms within individuals [29–31]. This specific question was addressed in a single-case study, which showed that a relapse in depression was preceded by rising autocorrelations in affective states [5]. These studies thus tentatively suggest that shifts in symptoms may indeed follow principles of complex systems. This implies that EWS could serve as a person-specific marker for vulnerability to future psychopathology. Before translating the existing evidence to clinical practice, however, these prior findings require substantiation on a larger scale. A first step in this direction is replicating the first prospective group-level study [4].

A second step that awaits empirical substantiation is to investigate whether EWS not only indicate the likelihood of shifts in the *severity* of symptoms, but also reveal the *type* of symptoms that will develop (e.g., depression, anxiety) [32]. This hypothesis proposes that the association between EWS in affective states and future increases in symptoms is particularly pronounced for affective states congruent with the psychopathological domain involved in the shift. For instance, EWS in feeling down might precede an upcoming shift towards depression, while EWS in feeling anxious might precede increases in anxiety [32]. If this hypothesis holds, EWS could inform clinicians about an individual’s vulnerability for specific disorders.

The current study was designed to further explore the role EWS in emerging psychopathology in two ways. First, building on the findings reported by van de Leemput et al. [4], we aimed to test whether EWS in affective states anticipate impending increases in depressive symptom severity. Second, we aimed to extend previous findings by examining whether EWS are predictive of the type of symptoms that will develop. We hypothesized that the association between symptom increases and EWS would be particularly pronounced for affective states congruent with the domain in which symptoms increased. The present paper specifically focuses on symptom increases, as opposed to decreases, because of our interest in emerging psychopathology and the possibility of early detection thereof. In view of a recent meta-analysis, which suggested that associations between autocorrelations and psychopathology might be attributable to individual differences in mean affect levels [28], we also investigated the associations between affect intensity (i.e., mean levels) and symptom increases. Similar to van de Leemput et al. [4], we analyzed a general population sample who provided ten daily affect ratings for 6 days using the ESM, resulting in time series that were used to compute EWS. Prior to the ESM and 1 year later, symptom severity was assessed. Participants who reported an increase in symptoms in one of the following domains were analyzed (hypothesized congruent affective states between brackets): depression (feeling down, listless), anxiety (feeling anxious, not relaxed), interpersonal sensitivity (feeling insecure, suspicious), and somatic complaints (feeling unwell).

## Methods

### Participants

Data were retrieved from the TwinssCan study [33], which comprised a subset of a registered cohort of twins from the general population (i.e., East Flanders Prospective Twin Survey) [34]. The study included *N* = 839 Caucasian twins aged between 15 and 34 years old. As particularly adolescents were invited to participate, most participants (*N* = 639) were between 15 and 18 years old. Complete baseline and follow-up SCL-90 ratings were available for 467 participants. We selected those participants who reported an increased symptom severity over time (i.e., baseline rating < follow-up rating) in one of the following domains of the Symptom Checklist-90 (SCL-90) [35]: depression, anxiety, somatic complaints, and interpersonal sensitivity. This criterion was adopted because we were interested in the development, rather than the remission, of symptoms. Symptom increases covered a broad range from minor impairments to clinically significant shifts—thereby allowing us to investigate symptom increases continuously [4].

To support the reliability of autocorrelation estimates, participants were excluded if less than 20 pairs of consecutive affect ratings were available. This resulted in four samples (*N* = 166, 184, 188, and 192). Since most participants reported increases in more than one domain, these samples were not unique in their composition. In total, the samples comprised 293 unique individuals, of whom 222 were included in more than one sample (see Additional file 1, Sample composition, Table S1). The TwinssCan study was approved by the Local Ethics Committee, and all subjects provided written informed consent. For minors, parents provided additional written consent.

### Experience sampling method

Affective states were assessed through the ESM, which is a structured diary technique suitable for assessing affect in daily life. The ESM involved questions on momentary affect, context, and behavior that were administered through PsyMates© [36]. Participants received a PsyMate (an electronic device) and an instruction about its usage at the beginning of the study. The PsyMate was programmed to emit a beep-signal at ten semi-random time intervals within 90-min blocks ranging from 7:30 AM to 10:30 PM for 6 consecutive days. The beep-signal alarmed participants to fill in a questionnaire concerning their current affective state. To support reliability and validity of these ratings, questionnaires that were completed more than 15 min after the beep were coded as missing [37]. Items were rated on a 7-point Likert scale, ranging from “not at all” to “very much.” Analyses were limited to a selection of seven items. These items were selected based on their congruency with one of the following psychopathological domains of interest (items between brackets): depression (feeling down, listless), anxiety (feeling anxious, not relaxed), interpersonal sensitivity (feeling insecure, suspicious), somatic complaints (feeling unwell; Table 1).

**Table 1:** Psychopathological domains and congruent affective states

Psychopathological domain (SCL-90)	Depression	Anxiety	Somatic complaints	Interpersonal sensitivity
ESM item	I feel down.	I feel anxious.	I feel unwell.	I feel insecure.
	I feel listless.	I do not feel relaxed.		I feel suspicious.

*Abbreviations: ESM* experience sampling method, *SCL-90* Symptom Checklist-90

### Assessment of psychopathology

Prior to the ESM as well as 1 year later, participants completed the Dutch version of the SCL-90 [35]. This questionnaire consists of 90 items and has good psychometric properties [38]. Items of the SCL-90 are rated on a 5-point Likert scale, ranging from “not at all” to “very often/always” (e.g., “During the last week, I felt empty”). Besides self-reported ratings of symptom severity in eight domains, the SCL-90 provides a global severity index (GSI) which can be used for screening purposes [38]. A GSI of 0.57 or higher has been suggested to optimally differentiate those who are at risk for psychiatric disorders from those who are not [39].

We selected the domains depression, anxiety, somatic complaints, and suspicion and interpersonal sensitivity (the latter two together referred to as interpersonal sensitivity) for analyses. Difference scores within these domains (i.e., follow-up score − baseline score) were computed to estimate changes in symptom severity. The domains depression and anxiety represent symptoms recognized in the corresponding clusters in the Diagnostic and Statistical Manual of Mental Disorders (DSM-V) [40]. Scores on the somatic complaints domain reflect non-specific physical discomfort. Finally, the interpersonal sensitivity domain incorporates feelings of interpersonal incompetence, suspicion, and paranoia. Other symptom clusters assessed by the SCL-90 were not selected because (i) the SCL-90 domain was not covered by ESM items (e.g., agoraphobia, sleeping problems, hostility, and insufficient thinking/feeling) or (ii) the psychopathological domain was non-specific (e.g., other complaints).

### Analyses

We specified a multilevel model^2^ to evaluate whether autocorrelations in affective states were related to increases in symptom severity. A similar model has been used by earlier studies [4, 23, 24, 26, 27, 41, 42]. In this model, the momentary affect rating (e.g., feeling down) of individual *i* belonging to twin-pair *j* at time *t* (affect_*tij*_) was predicted by the individual’s mean-centered affect rating at time *t-*1 (affect_*t* − 1*ij*_ − *μ*_*i*_), the individual’s increase in psychopathological symptoms from baseline to follow-up (*P*_*i*_), and an interaction term between symptom increases and the lagged person-mean centered affect rating (*P*_*i*_ × (affect_*t* − 1*ij*_ − *μ*_*i*_)). The model can be regarded as a multilevel extension of a standard autoregressive model: at the within-person level, we estimate an autoregressive model, and at the between-person level, we estimate the interaction between autoregressive coefficients and symptom increases.

In line with recommendations of [43] and earlier studies [41, 44–47], affect ratings at time *t-*1 were person-mean centered in order to ensure that parameters in the model were not affected by between-person differences in mean affect. Further, the first rating for each day was coded as missing in order to avoid autocorrelations that spanned a whole night rather than a few hours (cf. [24, 27]). We accounted for within-person and within-twin covariances in affect ratings by including a random intercept at the level of individuals and twins. Furthermore, a random effect for lagged affect ratings was estimated. In our multilevel model, *β*_3*ij*_ signifies the interaction effect of person-mean centered lagged affect ratings and symptom increases on actual affect ratings. This parameter thus illustrates the association between autocorrelating affective states and shifts in symptom severity.

Our first aim was to investigate whether EWS precede increases in depression. Therefore, analyses were restricted to increases in depressive symptoms, with affect items feeling down and feeling listless. van de Leemput et al. [4] used similar, yet less specific affective states, which were labeled according to their valence (high/low) and arousal (high/low). Our second aim was to explore whether autocorrelations in affective states are informative for the direction of shifts in symptom severity. This aim was addressed by applying the multilevel model^2^ to each of the four psychopathological domains and each of the seven affect items. The coefficients of the interaction effect between lagged affect ratings and increases in symptom severity on current affect ratings (*β*_3*ij*_) were standardized by multiplying the coefficients with the ratio of the standard deviation in the linear predictor (*P*_*i*_ × (affect_*t* − 1*ij*_ − *μ*_*i*_) ) and the standard deviation of the outcome (affect_*tij*_), respectively [48]. The standardized coefficients were then compared in order to assess for which affect items the relation between autocorrelations and increases in symptom severity was most pronounced. Comparisons were made across models (i.e., items) within domains and only for those items that were statistically significant [49]. The statistical significance of coefficients was adjusted according to Hochberg’s procedure [50], so that within each domain the type I error rate was .05.

The association between mean affect levels and symptom increases could not be retrieved directly from the multilevel model^2^. Hence, we employed linear regression analyses in order to examine whether mean affect levels are equally predictive of future symptom increases. Similar to van de Leemput et al. [4] and solely for the purposes of visualization, we re-ran the multilevel model outlined in footnote 2 with categorized instead of continuous change scores of symptom severity (*P*_*i*_). For each psychopathological domain, *P*_*i*_ scores were categorized based on tertile scores, resulting in three categories with approximately equal *N* (i.e., low, medium, and high symptom severity). Analyses were conducted in R (version 4.0).

## Results

Participants were grouped according to the SCL-90 domain in which they reported an increase in symptom severity. The resulting samples (*N* = 166–192) were similar regarding age, sex distribution, and SCL-90 domain scores at baseline and follow-up (Table 2; for details, see Additional file 1, Table S1). Based on the global severity index (GSI) of the SCL-90, the majority of participants scored below the clinical threshold (at baseline, 82–87%; at follow-up, 67–71%) [39]. Symptom increases exceeded the reliable change index reported by Schauenburg and Strack [39] in 26–31% of individuals. On average, participants completed 45.4 (76%) affect ratings.

**Table 2:** Sample characteristics

	Depression (*n* = 180)	Anxiety (*n* = 192)	Somatic complaints (*n* = 184)	Interpersonal sensitivity (*n* = 166)
No. male (%)	63 (35)	66 (34)	67 (36)	64 (39)
Age, mean (SD), years	17.9 (4.3)	18.0 (4.4)	17.9 (4.0)	17.9 (4.3)
No. of completed affect ratings, mean (SD)	45.3 (7.7)	45.4 (7.5)	45.4 (7.5)	45.4 (7.2)
Baseline SCL-90 domain score, mean (SD)	1.32 (0.33)	1.26 (0.34)	1.31 (0.37)	1.34 (0.34)
Follow-up SCL-90 domain score, mean (SD)	1.60 (0.51)	1.47 (0.51)	1.58 (0.56)	1.60 (0.50)
Baseline SCL-90 GSI, mean (SD)	0.36 (0.28)	0.39 (0.30)	0.40 (0.32)	0.36 (0.30)
Baseline no. above clinical threshold (%)^a^	24 (13)	35 (18)	32 (17)	25 (15)
Follow-up SCL-90 GSI, mean (SD)	0.49 (0.38)	0.48 (0.38)	0.49 (0.40)	0.51 (0.40)
Follow-up no. above clinical threshold (%)	55 (31)	56 (29)	57 (31)	54 (33)
Symptom increases above RCI (%)^b^	56 (31)	50 (26)	48 (26)	52 (31)
*ESM items, mean (SD)*				
Down	1.64 (0.78)	1.69 (0.80)	1.74 (0.85)	1.70 (0.83)
Listless	1.74 (0.86)	1.81 (0.88)	1.79 (0.87)	1.79 (0.90)
Anxious	1.39 (0.59)	1.45 (0.64)	1.45 (0.66)	1.44 (0.64)
Not relaxed	2.84 (1.18)	2.90 (1.20)	2.90 (1.19)	2.84 (1.21)
Unwell	2.73 (0.83)	2.78 (0.84)	2.79 (0.83)	2.77 (0.84)
Insecure	1.60 (0.79)	1.69 (0.84)	1.71 (0.85)	1.63 (0.83)
Suspicious	1.38 (0.56)	1.43 (0.61)	1.42 (0.61)	1.39 (0.58)

Samples were labeled according to the SCL-90 domain that was evaluated in subsequent analyses. Note that mean SCL-90 domain scores refer to the domain of interest (e.g., for depression, mean raw SCL-90 scores for the depression domain are presented). Means and standard deviations of ESM items were calculated within individuals within samples. *ESM* experience sampling method, *GSI* global severity index, *RCI* reliable change index, *SCL-90* Symptom Checklist 90, *SD* standard deviation

^a^Following the recommendations of Schauenburg and Strack [39], a GSI of 0.57 was adopted as clinical threshold

^b^The RCI equals 0.43 (for baseline GSI scores > .57) or 0.16 (for baseline GSI scores < 0.57; Schauenburg and Strack [39])

Our first aim was to investigate whether increases in depressive symptom severity are related to heightened autocorrelations in the affective states *feeling down* and *feeling listless*. For both *feeling down* and *feeling listless*, multilevel models did not reveal a significant interaction effect between symptom increases and person-mean centered affect ratings at time *t-*1 on affect ratings at time *t* (Table 3; *feeling down*: *β* = 0.02, SE_*β*_ = 0.02, *P* = .75, marginal *R*^2^ = 0.04, conditional *R*^2^ = 0.37^3^; *feeling listless: β* = 0.01, SE_*β*_ = 0.03, *P* = .75, marginal *R*^2^ = 0.04, conditional *R*^2^ = 0.40). In other words, autocorrelations in *feeling down* and *feeling listless* were not predictive of future symptom increases in adolescents from the general population.

**Table 3:** Standardized coefficients of interaction effects retrieved from multilevel models: EWS as predictors of symptom increases

Affect item	Depression (*n* = 180)		Anxiety (*n* = 192)		Somatic complaints (*n* = 184)		Interpersonal sensitivity (*n* = 166)	
	*β*	SE	*β*	SE	*β*	SE	*β*	SE
Down	0.02	0.02	0.04^†^	0.02	0.03	0.02	0.03	0.03
Listless	0.01	0.03	0.04	0.02	0.02	0.02	0.02	0.03
Anxious	0.02	0.03	0.05	0.03	0.01	0.03	0.04	0.03
Not relaxed	0.01	0.02	0.04^†^	0.02	0.00	0.02	0.01	0.02
Unwell	− 0.01	0.02	0.02	0.02	0.00	0.02	− 0.03	0.02
Insecure	0.04	0.02	0.02	0.02	0.02	0.02	0.05^†^	0.02
Suspicious	0.08^†^	0.03	0.05	0.03	0.06	0.03	0.12*	0.03

Coefficients refer to the standardized interaction effect of affect ratings at time *t-*1 and increases in psychopathological symptom severity on affect ratings at time *t.* This effect describes the relation between autocorrelations and symptom shifts. Note that other coefficients estimated by the multilevel model described in footnote 2, as well as the unstandardized estimates, are reported in Additional file 1, Tables S2 and S3. *SE* standard error

*Significant at *α* = 0.05, with *P* values adjusted according to Hochberg’s procedure

^†^Significant at *α* = 0.05, without multiple testing correction

**Fig. 1:**
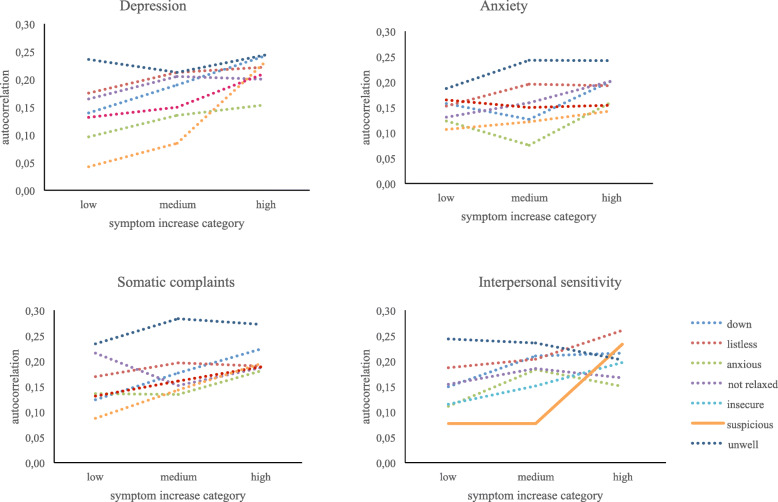
Association between autocorrelations in affective states and increases in symptom severity in different psychopathological domains (i.e., depression, anxiety, somatic complaints, and interpersonal sensitivity). SCL-90 scores were categorized solely for the purposes of visualization. After correcting for multiple testing, none of the trends was statistically significant (dotted lines), except for the association between increases in interpersonal sensitivity and EWS in feeling suspicious (solid line). Because visualization required the categorization of follow-up SCL-90 scores, the above figures approximate but not necessarily parallel the results in Table 3.

Our second aim was to examine whether EWS, reflected in autocorrelations in different affective states, are predictive of the direction of increases in symptoms. After correcting for an inflated type I error rate using Hochberg’s procedure [50], autocorrelations in feeling suspicious were predictive of larger symptom increases in the interpersonal sensitivity domain (*β* = 0.12, SE_*β*_ = 0.03, *P* < .01, marginal *R*^2^ = 0.04, conditional *R*^2^ = 0.42). The association between autocorrelating affective states and symptom increases was not statistically significant for other psychopathological domains (Table 3, Fig. 1). Other coefficients estimated in the multilevel model described in footnote 2—i.e., main effects of lagged person-mean centered affect ratings and symptom increases, respectively—as well as the unstandardized effects are denoted in Additional file 1, Tables S2 and S3. The variance in affect ratings (affect_*t*_) that could be explained by previous affect (affect_*t*-1_) and symptom increases—also referred to as the marginal *R*^2^—ranged from 0.02 to 0.06 (mean marginal *R*^2^ = 0.03, median = 0.04). Accounting for individual differences in autocorrelations and symptom increases raised the explained variance to 0.29 to 0.49 (mean conditional *R*^2^ = 0.38, median = 0.37).

Finally, we ran linear regression analyses to explore whether mean affect ratings were similarly predictive of symptom increases. Results were corrected for type I error inflation. Affect intensity (i.e., the average level of an affective state) was not predictive of future increases in symptoms after correcting for multiple testing (Additional file 1, Table S5). Not correcting for multiple testing yielded similar results.

### Post hoc analyses

The multilevel models adopted in the present study slightly deviate from the model described by van de Leemput et al. ([4]; analyses concerning general population sample). Specifically, we decided to examine the association between autocorrelations and *increases* in symptom severity, instead of the association between autocorrelations and *symptom severity at follow-up, corrected for baseline symptom severity* (see also Additional file 1, Post hoc analyses). To examine to what extent the results of the present study did or did not replicate those of van de Leemput et al. [4], we tested both procedures in both datasets. When applying the model described by van de Leemput et al. [4] to the current dataset, our results matched those reported in this earlier study: higher autocorrelations in affective states predicted greater symptom severity at follow-up (Additional file 1, Table S2). Similarly, when applying the model adopted in the present study to the dataset used by van de Leemput et al. [4], results confirmed those of the present study: autocorrelations in affective states were not related to symptom increases (Additional file 1, Table S3). Hence, the results of the both studies replicated perfectly in a second, independent dataset.

The discrepancy between both models in terms of results might be due to the fact that the inclusion of symptom severity at baseline as a predictor (cf. van de Leemput et al. [4]) corrects the *outcome* of the model (affect at time *t*)—but not another *predictor* in the model, namely follow-up symptom severity—for baseline symptoms. Hence, including baseline symptoms does not mean that follow-up symptoms can be considered indicative of change scores. We therefore consider the model used in the present study—denoted in footnote 2—to be better suited for investigating the association between autocorrelations and symptom increases than the model reported earlier ([4], analyses concerning general population sample).

In both datasets, the correlation between symptom severity at baseline and follow-up was high (current dataset: *r* = 0.79–0.86, depending on the subsample; dataset van de Leemput et al. [4]: *r* = 0.68). As a result, the association between autocorrelations and symptoms at follow-up (with baseline symptoms included as a predictor) that was found in both datasets might in fact reflect an association between autocorrelations and concurrent symptom severity. This indeed appeared to be the case both for the dataset provided by van de Leemput et al. ([4]; Additional file 1, Table S3) and for the current dataset (Additional file 1, Table S4). In the latter, autocorrelations in *feeling suspicious* were most strongly related to baseline symptom severity (Additional file 1, Table S4). This generic effect was followed by domain-specific effects, showing that the association between autocorrelations and baseline symptom severity was particularly pronounced for congruent combinations of affective states and psychopathological domains (Table 1; Additional file 1, Table S4). Affect intensity (i.e., the mean level of an affective state) was also predictive of higher symptom severity at baseline (Additional file 1, Table S5), but these associations did not differentiate according to the type of affective state or psychopathological domain involved.

## Discussion

This study found that increases in psychopathology after 1 year were generally not associated with baseline autocorrelations in negative affective states. The only exception was a significant association between elevated autocorrelations (EWS) in *feeling suspicious* and increases in interpersonal sensitivity. It follows that we found only marginal support for EWS as predictors of symptom shifts after 1 year, and no support for the hypothesis that EWS could signal the *type* of symptoms about to develop. Post hoc analyses, which included a second dataset provided by van de Leemput et al. [4], revealed an identical pattern of results across two datasets: autocorrelations are predictive of *concurrent* depressive symptoms, but not of *future* depressive symptoms. Thus, it seems that group-level designs (present study and van de Leemput et al. [4]) do not lead to the same conclusion as individual-level studies that reported rising trends in autocorrelations in individuals who were about to relapse into depression [5, 51]. Methodological differences between both designs likely account for this and provide useful guidance for further studies into EWS for psychopathological shifts.

### Paradox between group- and individual-level studies

The majority of earlier studies concerning complex system principles in psychopathology involved either simulated data [52, 53] or intensive longitudinal data collected within individuals [11, 13]. Examples of the latter include two earlier case studies that adopted an experience sampling design, which allows for monitoring symptom shifts as well as affect dynamics (including autocorrelations; EWS) as they evolve within individuals. These studies found rising trends in the autocorrelation of negative mental states several weeks before a relapse in patients suffering from depression [5, 51]. The present findings suggest that these *within-individual* processes implied by complex systems principles—namely, accumulating instability prior to symptom shifts—hardly create differences *between individuals*—namely, higher autocorrelations in those who report higher symptom shifts [30]. A likely explanation for this concerns the different timescales that were considered. While within-individual designs investigated EWS over the course of several weeks [5, 51], between-individual designs (including the present study) investigated EWS 1 to 3 years prior to symptom shifts [4, 21]. Taken together, these studies provide novel insight in the timescale at which critical slowing down manifests, and also suggest that this timescale may differ between psychopathological domains. For instance, depressive symptoms might be characterized by relatively fast dynamics, suggesting that EWS might only appear in the weeks prior to a symptom shift [5, 51], while interpersonal sensitivity might show slower dynamics, meaning that EWS could already manifest 1 year in advance. Further research is needed in order to establish the timescale at which EWS unfold within individuals with varying psychopathological complaints. Such research could also address the size of symptom shifts for which EWS are relevant. In conclusion, the utility of complex systems principles to psychopathology may be restricted to specific circumstances, e.g., characterized by specific time intervals, types of psychopathology, and sizes of shifts. It requires large-scale within-individual research, where EWS and symptom shifts are monitored in real-time in a considerable number of individuals, to delineate under what circumstances EWS could be informative of future symptom progression.

### Autocorrelations in momentary affective states as signs of current symptomatology

While associations between autocorrelations and *future symptom increases* were lacking for almost all affective states/psychopathological domains, post hoc analyses showed that associations between autocorrelations in affective states and *symptom severity at baseline* were present across multiple domains. This pattern replicated in an independent dataset provided by van de Leemput et al. [4]. The extent to which momentary affective states carry over from one moment to the next thus appeared to be related to concurrent levels of psychopathological severity. Correspondingly, earlier studies found that emotional inertia, indexed by elevated autocorrelations in affective states, is cross-sectionally related to psychopathology [20, 24, 26, 27]. From an emotion-regulation perspective, emotional inertia signals what is labeled in this literature as rigidity [21, 27]. That is, individuals with high levels of inertia may “get stuck” in negative affective states and hence be vulnerable for symptoms of psychopathology [27]. However, inertia also coincides with heightened intensity of negative affect, raising the question to what extent their respective associations with psychopathology are shared. According to a recent meta-analysis, inertia and affect intensity show little overlap, with less than 6% of shared variance [28]. Nevertheless, the association between inertia and depressive symptoms was weak and therefore dropped to “drastic non-significance” after accounting for affect intensity [28]. This might be explained by the small effect size of inertia on depressive symptoms. Hence, it seems that for predicting psychopathology, more parsimonious metrics of affective experience (affect intensity) suffice. Yet, inertia could still provide unique insight in the processes that give rise to psychopathology. Present findings suggest, for instance, that the association between inertia (autocorrelations) and psychopathology might show domain specificity. Specifically, symptoms of depression, anxiety, and interpersonal sensitivity were more associated with autocorrelations in affective states that were a priori considered to cover that domain (Table 1; e.g., for depression, symptom severity was most strongly associated to autocorrelations in feeling suspicious, down, and listless, while for anxiety, symptom severity was associated to autocorrelations in feeling suspicious and anxious). In contrast, mean levels were predictive of concurrent symptom severity regardless of the affective state or psychopathological domain assessed. Speculatively, this could mean that the intensity of negative affective states is informative of global symptom severity, while the dynamics of those affective states (reflected in their autocorrelations) might reveal the types of symptoms that are present. Further disentangling the unique effects of affect dynamics (including EWS) and affect intensity, respectively, in the context of psychopathology should be considered an important avenue for further research.

### Strengths, limitations, and future directions

EWS have been suggested to hold great promise for clinical practice: if found to be predictive of future psychopathology, EWS could help us to foresee and possibly prevent illness progression. This means that clinical advancements would no longer be hindered by limited understanding of the complex mechanisms that underlie onset or progression of psychopathology. To examine the value of this promise, comprehensive and critical investigation of the utility of EWS to psychopathology is necessary. We therefore aimed to replicate and extend the first—and to date, only—study that provided empirical, group-level support for the association between EWS and symptom increases [4]. Across two datasets with a similar sample, experience sampling protocol and assessment of psychopathology, we found an identical pattern of results, which considerably strengthens our conclusions. However, a few limitations require attention. First, we were selectively interested in symptom increases—and the possibility that EWS might foresee such increases. Analyses were therefore restricted to individuals who reported symptom increases, which may have created an endogenous selection bias. This bias arises when the findings obtained in a subsample that was selected based on a collider variable (symptom increases) do not match findings that would have been obtained in the full sample (i.e., individuals with both increases and decreases in symptoms) [54, 55]. Our findings are therefore only informative of individuals whose symptom increase. However, they are precisely the individuals of interest, as this study focused on EWS as prospective indicators of the onset or progression (i.e., increase) of psychopathology rather than recovery (for studies on the latter, see ref. [8, 15]). A second limitation is that neither the reliability of individual experience sampling items nor the reliability of autocorrelations could be verified, which highlights the need for psychometric reliability metrics suitable for experience sampling data. Finally, the present group-level design could only indirectly investigate the relation between EWS and psychopathology and, like earlier studies [4, 11, 14, 21, 24, 26, 27, 42, 56], does not allow for within-individual inferences. A more direct evaluation of the hypotheses that follow from a complex systems perspective on psychopathology requires designs where individuals who are at increased risk for psychopathology prospectively monitor their mental state for a prolonged time period (e.g., several months) during which a shift is likely to occur [5]. Such designs could verify whether, within individuals, sudden shifts in symptom severity are preceded by rising patterns in EWS. Real-time monitoring of EWS and symptom shifts allows addressing critical research questions that will ultimately determine their usefulness to clinical practice. Such questions relate to the timing of EWS, the size and type of symptom shifts that can be anticipated, the sensitivity and specificity of EWS in the context of psychopathology, and the absolute thresholds in EWS that may inform clinical decision-making.

## Conclusions

Empirical investigation of complex systems principles in psychopathology is still in its infancy. Hence, the present study should be considered a first step towards investigating whether complex systems principles apply to psychopathology. Across two datasets, we found that autocorrelations in affective states are related to concurrent symptoms of depression, but not to increases in those symptoms after 1 year*.* Yet, increases in interpersonal sensitivity were preceded by heightened autocorrelations, which suggests that the association between early warning signals and psychopathology might depend on the domain wherein symptoms increase. Further research is needed to delineate when, for what type of symptoms, and for what size of shifts, early warning signals are predictive of future psychopathology. This requires monitoring early warning signals and symptoms in real-time, thereby allowing for a more direct evaluation of the inferences that follow from a complex systems perspective on psychopathology.

## Supplementary information

**Additional file 1.** Reports details on the sample composition, and particularly, the overlap between samples; details concerning the main analyses (e.g. effects that were not of primary interest; unstandardized effects); and results from post-hoc analyses where the models of the study by van de Leemput et al. [4] and models of the current study are compared and fitted to both datasets.

## Data Availability

The datasets analyzed during the current study are not publicly available due to the possibility to identify participants based on their clinical and experience sampling data (European law).

## Abbreviations

ESM: Experience sampling method
EWS: Early warning signals
GSI: Global severity index
SCL-90: Symptom Checklist-90
SD: Standard deviation
SE: Standard error

## Footnotes

1 Throughout the rest of this article, EWS will refer to autocorrelations.

2 Multilevel model to investigate the association between autocorrelation in affective states and shifts in psychopathology:

$$ {\mathrm{affect}}_{tij}={\beta}_{0 ij}+{\beta}_{1 ij}\left({\mathrm{affect}}_{t-1 ij}-{\mu}_i\right)+{\beta}_{2 ij}{P}_i+{\beta}_{3 ij}\left({P}_i\times \ast \left({\mathrm{affect}}_{t-1 ij}-{\mu}_i\right)\right)+{\varepsilon}_{ij} $$

This equation translates to R code as follows: affect_*t*_~(affect_*t* − 1_ − *μ*_*i*_) + *P* + (*P* × (affect_*t* − 1_ − *μ*_*i*_)) + (1 + (affect_*t* − 1_ − *μ*_*i*_) | twinID/ID), where twinID denotes the factor that distinguishes between twin pairs and ID represents the individual.

3 Marginal *R*^2^ reflects the variance explained by the fixed effects; conditional *R*^2^ reflects the variance explained by the full model (i.e., fixed and random effects).
