# Supplementary material for: Early warning signals in psychopathology: what do they tell?
Source: BMC Med. 2020 Oct 14;18:269. doi: 10.1186/s12916-020-01742-3 (PMC7557008; doi:10.1186/s12916-020-01742-3)
Supplement: Supplementary file 1 — Additional file 1. Reports details on the sample composition, and particularly, the overlap between samples; details concerning the main analyses (e.g. effects that were not of primary interest; unstandardized effects); and results from post-hoc analyses where the models of the study by van de Leemput et al. [4] and models of the current study are compared and fitted to both datasets. [file 12916_2020_1742_MOESM1_ESM.docx]

**Additional file 1.**

**Sample composition**

Due to attrition, SCL-90 data at follow-up were only available for a subset of individuals, which reduced our sample size from N=839 to N=467. Compared to individuals with complete SCL-90 data, individuals with incomplete data (N=372) were younger (t(837)=3.27, P<.01, d=.23) and more likely to be male (χ^2^(1)=22.71, P<.01). Individuals with complete versus incomplete SCL-90 data did not differ in total SCL-90 score at baseline (t(808)=0.56, P=.57, d=.04). Of the 467 individuals with complete baseline and follow-up SCL-90 ratings, we selected those individuals who reported increased symptom severity from baseline to follow-up. This resulted in four samples of N=230 (depression), N=252 (anxiety), N=243 (somatic complaints), and N=220 (interpersonal sensitivity). Within these samples, individuals with at least 20 consecutive affect ratings were selected, resulting in samples of N=180 (depression), N=192 (anxiety), N=184 (somatic complaints), and N=166 (interpersonal sensitivity). Because most participants reported increased symptom severity in more than one symptom cluster, these samples were not unique in their composition. That is, any two samples showed an overlap ranging from 110 to 124 individuals (table S1). Of the 293 unique individuals that made up the samples, 64 were present in each sample. These participants did not differ from other participants with respect to age (t(291)=0.76, P=.45, d=.11) or gender (χ^2^(1)=0.74, P=.39), but did report higher total SCL-90 scores at baseline (t(291)=3.46, P<.01, d=.50). Because participants were retrieved from a registered cohort of twins, they were not independent from each other. This dependency was accounted for in multilevel models. The sample of 293 unique individuals consisted of 170 (58.0%) twins, 116 (39.6%) genetically unrelated participants, 5 (1.7%) triplets and 2 (0.7%) siblings.

**Table S1. Overlap between samples**

|  | Depression  (N=180) | Anxiety  (N=192) | Somatic complaints  (N=184) |
| --- | --- | --- | --- |
| Anxiety (N=192) | 124 |  |  |
| Somatic complaints (N=184) | 112 | 122 |  |
| Interpersonal sensitivity (N=166) | 119 | 113 | 110 |

Numbers denote the number of individuals that were present in both the sample denoted in the row and the sample denoted in the column.

| Affect item | Depression  (n=180) | | | | Anxiety  (n=192) | | | | Somatic complaints  (n=184) | | | | Interpersonal sensitivity  (n=166) | | | |
| --- | --- | --- | --- | --- | --- | --- | --- | --- | --- | --- | --- | --- | --- | --- | --- | --- |
|  | Lagged affect  β_1ij_ | SE | Sympt. increase β_2ij_ | SE | Lagged affect  β_1ij_ | SE | Sympt. increase β_2ij_ | SE | Lagged affect  β_1ij_ | SE | Sympt. increase β_2ij_ | SE | Lagged affect  β_1ij_ | SE | Sympt. increase β_2ij_ | SE |
| Down | 0.14** | 0.02 | 0.11 | 0.04 | 0.11** | 0.02 | 0.15** | 0.04 | 0.12** | 0.02 | 0.13* | 0.04 | 0.13** | 0.03 | 0.17** | 0.05 |
| Listless | 0.16** | 0.02 | 0.10 | 0.05 | 0.13** | 0.02 | 0.12* | 0.04 | 0.14** | 0.02 | 0.10 | 0.04 | 0.16** | 0.03 | 0.13* | 0.04 |
| Anxious | 0.10** | 0.03 | 0.09 | 0.04 | 0.08** | 0.02 | 0.15** | 0.04 | 0.12** | 0.02 | 0.15** | 0.04 | 0.09** | 0.03 | 0.12* | 0.04 |
| Not relaxed | 0.15** | 0.02 | 0.05 | 0.04 | 0.11** | 0.02 | 0.05 | 0.04 | 0.16** | 0.02 | 0.02 | 0.04 | 0.14** | 0.02 | 0.04 | 0.04 |
| Unwell | 0.18** | 0.02 | 0.14* | 0.05 | 0.16** | 0.02 | 0.13* | 0.05 | 0.20** | 0.02 | 0.14* | 0.05 | 0.19** | 0.02 | 0.07 | 0.05 |
| Insecure | 0.11** | 0.02 | 0.05 | 0.04 | 0.12** | 0.02 | 0.09 | 0.04 | 0.12** | 0.02 | 0.05 | 0.04 | 0.10** | 0.02 | 0.08 | 0.04 |
| Suspicious | 0.06* | 0.03 | 0.08 | 0.04 | 0.08** | 0.02 | 0.14** | 0.04 | 0.09** | 0.02 | 0.14** | 0.04 | 0.04 | 0.03 | 0.13* | 0.04 |

**Table S2. Standardized coefficients of main effects retrieved from multilevel models: EWS as predictors of symptom increases**

Coefficients refer to the standardized effects of (1) person-mean centered affect ratings at time t-1 (β_1ij_) and (2) increases in psychopathological symptom severity (β_2ij_) on affect ratings at time t, accounting for baseline symptom severity (model: affect_tij_ = β_0ij_ + β_1ij_ (affect_t-1ij_ - µ_i_) + β_2ij_ P_i_ + β_3ij_ (P_i_ * (affect_t-1ij_ - µ_i_)) + ε_ij_). The interaction effects of affect ratings at time t-1 and increases in psychopathological symptom severity on affect ratings at time t (β_3ij_) are reported in the main manuscript (table 3). SE: standardized standard error; Sympt.: symptom. *Significant at α=0.05, **Significant at α=0.005, with P-values adjusted according to Hochberg’s procedure.

**Table S3. Unstandardized coefficients of effects retrieved from multilevel models: EWS as predictors of symptom increases**

| Affect item | Depression  (n=180) | | | | | | Anxiety  (n=192) | | | | | | Somatic complaints  (n=184) | | | | | | Interpersonal sensitivity  (n=166) | | | | | |  |
| --- | --- | --- | --- | --- | --- | --- | --- | --- | --- | --- | --- | --- | --- | --- | --- | --- | --- | --- | --- | --- | --- | --- | --- | --- | --- |
|  | B_1ij_ | SE | B_2ij_ | SE | B_3ij_ | SE | B_1ij_ | SE | B_2ij_ | SE | B_3ij_ | SE | B_1ij_ | SE | B_2ij_ | SE | B_3ij_ | SE | B_1ij_ | SE | B_2ij_ | SE | B_3ij_ | SE | |
| Down | 0.17 | 0.03 | 0.36 | 0.14 | 0.05 | 0.05 | 0.14 | 0.02 | 0.60 | 0.17 | 0.12 | 0.06 | 0.15 | 0.03 | 0.46 | 0.15 | 0.07 | 0.06 | 0.16 | 0.03 | 0.72 | 0.19 | 0.10 | 0.08 | |
| Listless | 0.20 | 0.03 | 0.33 | 0.16 | 0.02 | 0.06 | 0.16 | 0.02 | 0.49 | 0.18 | 0.11 | 0.06 | 0.17 | 0.03 | 0.39 | 0.17 | 0.04 | 0.06 | 0.20 | 0.03 | 0.58 | 0.20 | 0.05 | 0.08 | |
| Anxious | 0.12 | 0.03 | 0.24 | 0.10 | 0.04 | 0.06 | 0.10 | 0.03 | 0.48 | 0.13 | 0.12 | 0.07 | 0.14 | 0.03 | 0.44 | 0.11 | 0.02 | 0.05 | 0.11 | 0.03 | 0.39 | 0.14 | 0.11 | 0.07 | |
| Not relaxed | 0.18 | 0.02 | 0.20 | 0.17 | 0.03 | 0.05 | 0.14 | 0.02 | 0.25 | 0.19 | 0.13 | 0.06 | 0.19 | 0.02 | 0.10 | 0.17 | 0.01 | 0.05 | 0.16 | 0.02 | 0.23 | 0.22 | 0.02 | 0.06 | |
| Unwell | 0.24 | 0.03 | 0.48 | 0.17 | -0.02 | 0.06 | 0.21 | 0.02 | 0.55 | 0.19 | 0.08 | 0.07 | 0.26 | 0.03 | 0.48 | 0.17 | 0.00 | 0.06 | 0.25 | 0.03 | 0.29 | 0.21 | -0.09 | 0.08 | |
| Insecure | 0.13 | 0.03 | 0.15 | 0.14 | 0.10 | 0.05 | 0.14 | 0.02 | 0.36 | 0.17 | 0.06 | 0.07 | 0.15 | 0.03 | 0.17 | 0.16 | 0.05 | 0.06 | 0.12 | 0.03 | 0.34 | 0.18 | 0.14 | 0.07 | |
| Suspicious | 0.07 | 0.03 | 0.20 | 0.11 | 0.17 | 0.07 | 0.10 | 0.03 | 0.42 | 0.12 | 0.12 | 0.07 | 0.11 | 0.03 | 0.39 | 0.11 | 0.11 | 0.06 | 0.05 | 0.03 | 0.42 | 0.14 | 0.32 | 0.07 | |

Coefficients refer to the unstandardized effects of (1) person-mean centered affect ratings at time t-1 (B_1ij_), (2) increases in psychopathological symptom severity (B_2ij_), and (3) the interaction between person-mean centered affect ratings and increases in psychopathological symptom severity (B_3ij_) on affect ratings at time t (model: affect_tij_ = B_0ij_ + B_1ij_ (affect_t-1ij_ - µ_i_) + B_2ij_ P_i_ + B_3ij_ (P_i_ * (affect_t-1ij_ - µ_i_)) + ε_ij_). SE: unstandardized standard error. Due to limited space, the statistical significance of coefficients could not be displayed. However, this significance is reported in table S2 (B_2ij_ and B_3ij_) and table 2 (main manuscript, B_3ij_).

**Post-hoc analyses**

The primary aim of this study was to test whether EWS – operationalized as the autocorrelations in momentary affective states – precede increases in symptoms. We originally addressed this aim through a multilevel model similar to the model described by Van de Leemput *et al.* (2014; analyses concerning the general population sample). In this model, outlined in formula 1, the affect rating of individual *i* belonging to family *j* at time *t* (${affect}_{tij}$) is predicted by the same individual’s mean-centered affect rating at time *t-1* (${affect}_{t-1ij}-\mu_{i}$), his/her symptom severity at baseline (${Pb}_{i})$, his/her symptom severity at follow-up (${Pf}_{i}$), and an interaction effect between symptom severity at follow-up and the lagged person mean-centered affect rating ($\beta_{4ij}$).

$${affect}_{tij}=\beta_{0ij}+\beta_{1ij}\left( {affect}_{t-1ij}-\mu_{i} \right)+{\beta_{2ij}Pb}_{i}+\beta_{3ij}{Pf}_{i}+\beta_{4ij}\left( {Pf}_{i}\times\left( {affect}_{t-1ij}-\mu_{i} \right) \right)+ \varepsilon_{tij}$$

***Formula 1.* Multilevel model that tests the association between the autocorrelation in momentary affective states and symptom severity at follow-up.**

Thanks to an anonymous reviewer, we realized that the coefficient denoting the interaction effect in this model ($\beta_{4ij}$) does not test whether elevated autocorrelations precede *increases* in symptoms – as hypothesized from a complex systems perspective on psychopathology – but rather tests the relation between autocorrelations in affective states and *symptom severity at follow up*. We therefore adapted the model above to accommodate our questions and hypotheses (Formula 2; see analysis section in the main text for a description of this model).

$${affect}_{tij}= \beta_{0ij}+\beta_{1ij}\left( {affect}_{t-1ij}-\mu_{i} \right)+ \beta_{2ij}P_{i}+ \beta_{3ij}\left( P_{i}*\left( {affect}_{t-1ij}-\mu_{i} \right) \right)+ \varepsilon_{tij}$$

***Formula 2.* Multilevel model that tests the association between the autocorrelation in momentary affective states and increases in symptoms.** In this model, $P_{i}$ denotes the increase in symptoms from baseline to follow-up.

In order to test how this adaptation of the model affected our results, both models were run. Results obtained from the second model (formula 2) – which addressed our primary aim – revealed that elevated autocorrelations preceded increases in interpersonal sensitivity, but not increases in other psychopathological domains (*i.e.* depression, anxiety, somatic complaints; see table 3 in main text). Results obtained from the first model (formula 1) – which mirrored the model reported by Van de Leemput *et al.* (2014) – showed that autocorrelations in momentary affective states were positively related to the severity of symptoms of depression, anxiety, and interpersonal sensitivity at follow up (see table S4 below).

Post-hoc, we examined whether the discrepant results of both models replicated in the dataset analyzed by Van de Leemput *et al.* (2014). This dataset consists of 621 individuals from the general population who provided ratings of their momentary affective state ten times a day for six consecutive days. At baseline and at four follow-up assessments – which were scheduled three months apart – participants completed the Symptom Checklist-90 (SCL-90). Symptom severity at follow-up was calculated as the average score of these four follow-up assessments. In line with the analyses reported by Van de Leemput *et al.* (2014), affect ratings were aggregated according to their valence and arousal. The resulting scores will be referred to as feeling down (low valence and arousal) and feeling anxious (low valence, high arousal). Further, analyses were restricted to the SCL-90 domain depression. Complete data were available for 533 participants. For a detailed description of the sample and methods, see Van de Leemput *et al.* (2014).

The results of these supplementary analyses confirmed our initial findings. Specifically, autocorrelations in feeling down and feeling anxious were predictive of higher depressive symptoms at follow-up, corrected for baseline (formula 1, cf. results of Van de Leemput *et al.* (2014)) but not of higher *increases* in depressive symptoms from baseline to follow-up (formula 2; table S5). The fact that this pattern emerged in two independent datasets led us to further explore the discrepancies between both models (formula 1 and 2, respectively). We hypothesized that the association between autocorrelations and symptoms at follow-up, corrected for baseline (formula 1), might in fact be accounted for by the association between autocorrelations and symptoms at baseline (which was not sufficiently controlled for in the model). In both datasets, this hypothesis was confirmed (table S5).

**Table S2. Standardized coefficients of interaction effects retrieved from multilevel models: EWS as predictors of future symptoms**

| Affect item | Depression  (n=180) | Anxiety  (n=192) | Somatic complaints (n=184) | Interpersonal sensitivity  (n=166) |
| --- | --- | --- | --- | --- |
| Down | 0.10** | 0.06^†^ | 0.04 | 0.07^†^ |
| Listless | 0.09* | 0.06^†^ | 0.02 | 0.05 |
| Anxious | 0.08^†^ | 0.09* | 0.04 | 0.06 |
| Not relaxed | 0.04 | 0.04^†^ | 0.00 | 0.02 |
| Unwell | 0.04 | 0.01 | 0.01 | 0.01 |
| Insecure | 0.07* | 0.05^†^ | 0.02 | 0.08* |
| Suspicious | 0.18** | 0.10* | 0.07^†^ | 0.15** |

Coefficients refer to the standardized interaction effect of affect ratings at time *t-1* and psychopathological symptom severity at follow-up on affect ratings at time *t*, accounting for baseline symptom severity (formula 1)*.* This effect describes the relation between autocorrelations and future symptom severity.

*Significant at α=0.05, **Significant at α=0.005, with P-values adjusted according to Hochberg’s procedure; ^†^Significant at α=0.05, without multiple testing correction.

**Table S3. Standardized coefficients of interaction effects retrieved from two multilevel models in two datasets**

|  | Increases in symptoms of depression | | | | Severity of symptoms of depression at follow-up | | | | Severity of symptoms of depression at baseline | | | |
| --- | --- | --- | --- | --- | --- | --- | --- | --- | --- | --- | --- | --- |
|  | Original study  (n=180) | | Replication study  (n=533) | | Original study  (n=180) | | Replication study  (n=533) | | Original study  (n=180) | | Replication study  (n=533) | |
|  | β | B | β | B | β | B | β | B | β | B | β | B |
| Down | 0.02 | 0.05 | 0.00 | -0.01 | 0.10** | 0.12** | 0.13* | 0.07* | 0.12** | 0.23** | 0.13* | 0.06* |
| Anxious | 0.02 | 0.04 | -0.02 | -0.04 | 0.08* | 0.09* | 0.11* | 0.06* | 0.09* | 0.16* | 0.16** | 0.08** |

Original study refers to the data used in the main text of this paper, which were obtained from the TwinssCan study (37); replication study refers to the general population sample reported by Van de Leemput *et al.* (2014). The first columns (‘increases in symptoms of depression’) denote the interaction effect of increases in symptoms of depression and lagged person-mean centered affect ratings on actual affect ratings (formula 2). This effect describes the association between autocorrelations in momentary affective states and symptom increases. The middle columns (‘severity of symptoms of depression at follow-up’) denote the interaction effect of depressive symptom severity at follow-up and lagged person-mean centered affect ratings on actual affect ratings (formula 1). This effect describes the association between autocorrelations in momentary affective states and symptom severity at follow-up. Finally, the last columns (‘severity of symptoms of depression at baseline’) denote the interaction effect of depressive symptom severity at baseline and lagged person-mean centered affect ratings on actual affect ratings. To remain consistent with the results reported by Van de Leemput *et al.* (2014), both standardized (β) and non-standardized (B) coefficients are denoted.

*Significant at α=0.05; **Significant at α=0.005

**Table S4. Standardized coefficients of interaction effects retrieved from multilevel models: EWS as predictors of current symptoms**

| Affect item | Depression  (n=180) | Anxiety  (n=192) | Somatic complaints (n=184) | Interpersonal sensitivity  (n=166) |
| --- | --- | --- | --- | --- |
| Down | 0.12** | 0.04 | 0.02 | 0.06^†^ |
| Listless | 0.11** | 0.05^†^ | 0.01 | 0.06^†^ |
| Anxious | 0.09* | 0.08* | 0.04 | 0.05 |
| Not relaxed | 0.04* | 0.02 | -0.01 | 0.02 |
| Unwell | 0.06* | 0.00 | 0.01 | 0.03 |
| Insecure | 0.06* | 0.05^†^ | 0.01 | 0.06^†^ |
| Suspicious | 0.15** | 0.09* | 0.05^†^ | 0.10* |

Coefficients refer to the standardized interaction effect of affect ratings at time *t-1* and psychopathological symptom severity at baseline on affect ratings at time *t.* This effect describes the relation between autocorrelations and future symptom severity.

*Significant at α=0.05, **Significant at α=0.005, with P-values adjusted according to Hochberg’s procedure; ^†^Significant at α=0.05, without multiple testing correction.

**Table S5. Affect intensity as a predictor of symptom increases, future symptom severity, and current symptom severity**

| Affect item | Depression  (n=180) | | | Anxiety  (n=192) | | | Somatic complaints  (n=184) | | | Interpersonal sensitivity  (n=166) | | |
| --- | --- | --- | --- | --- | --- | --- | --- | --- | --- | --- | --- | --- |
|  | Symptom increases | Future symptom severity | Current symptom severity | Symptom increases | Future symptom severity | Current symptom severity | Symptom increases | Future symptom severity | Current symptom severity | Symptom increases | Future symptom severity | Current symptom severity |
| Down | 0.03 | 0.25** | 0.19** | 0.03 | 0.24** | 0.17** | 0.05 | 0.23** | 0.16** | 0.04 | 0.24** | 0.17** |
| Listless | 0.02 | 0.17** | 0.12** | 0.01 | 0.19** | 0.15** | 0.03 | 0.19** | 0.13** | 0.04 | 0.17** | 0.11** |
| Anxious | 0.04 | 0.20** | 0.13** | 0.03 | 0.25** | 0.18** | 0.06 | 0.20** | 0.12** | 0.03 | 0.17** | 0.12** |
| Not relaxed | 0.01 | 0.09* | 0.07* | 0.00 | 0.13** | 0.10** | 0.01 | 0.05 | 0.04 | 0.01 | 0.09^†^ | 0.07* |
| Unwell | 0.04 | 0.20** | 0.14** | 0.02 | 0.21** | 0.15** | 0.03 | 0.18** | 0.12** | 0.01 | 0.14** | 0.11** |
| Insecure | 0.01 | 0.14** | 0.10** | 0.00 | 0.20** | 0.15** | 0.01 | 0.11* | 0.08* | 0.02 | 0.14** | 0.10** |
| Suspicious | 0.03 | 0.13** | 0.08** | 0.04 | 0.22** | 0.15** | 0.07 | 0.21** | 0.12** | 0.03 | 0.13** | 0.08* |

Numbers denote standardized coefficients retrieved from linear regressions where mean affect ratings predicted, respectively, symptom severity at follow-up while correcting for symptom severity at baseline (*i.e.* symptom increases), symptom severity at follow-up (*i.e.* future symptom severity), and symptom severity at baseline (*i.e.* current symptom severity).

*Significant at α=0.05, **Significant at α=0.005, with P-values adjusted according to Hochberg’s procedure; ^†^Significant at α=0.05, without multiple testing correction.
